# Solving the Mystery of Myelodysplasia

**DOI:** 10.1371/journal.pmed.0050040

**Published:** 2008-02-12

**Authors:** Jerald Radich

## Abstract

The author reviews key research on the biology underlying treatment response in myelodysplasia, including a new study in*PLoS Medicine*.

Myelodysplasia (MDS) is a clonal hematopoietic malignancy as stubborn in revealing its pathogenesis as it is in responding to treatment. The disease presents with cytopenia of any or all of the three hematopoietic lineages (red blood cells, platelets, and white blood cells), manifesting clinically as fatigue, bleeding, and infectious disorders. While the disease occurs in only five per 100,000 people, its incidence rises steeply with age, reaching about 20–40 per 100,000 at age 70 years and beyond. Thus, as the population ages, the impact of MDS on the health care system will grow.

The natural history of MDS is highly variable. By definition, cases present with low blood counts. Without treatment (and often, despite treatment), patients will embark on one of two paths of disease course, both inevitably fatal. Many will have progressive cytopenias, and eventually succumb to bleeding and infection complications. Other patients progress to acute myeloid leukemia (AML). Unlike “de novo” AML (where the patient presents with leukemia without an antecedent history of a hematopoietic disorder), which can be cured with high-dose combination chemotherapy in about 25% of cases, the “secondary” AML that arises from MDS is incurable by chemotherapy [1].

## Classification and Prognosis

The diagnosis of MDS requires the bone marrow evidence of dysplasia (abnormal appearing cells) in at least one of the hematopoietic cell lineages. Approximately 50% of cases will have chromosomal abnormalities. The appearance of bone marrow “blasts” (immature progenitor cells) can be normal in many cases, if the blasts represent less than 5% of total bone marrow cells; increasing blasts herald a progression towards AML. Sensitive flow cytometric analysis of cell surface antigens often shows a population of cells displaying an abnormal constellation of antigens, further evidence of disturbed hematopoiesis.

Diagnostic and classification schemes are based on the extent of cytopenia, cytogenetic abnormalities, blast percentages, and other morphological features. The older French, American, and British (FAB) diagnostic system divided the disease into refractory anemia (RA), where blast counts were <5%; RA with ringed sideroblasts (RARS); and two classes with increasing blast counts, RA with excessive blasts of >5% (RAEB), and with excessive blasts (>20%) in “transition” to AML (RAEBT). Secondary AML arising from MDS was defined as a marrow blast count of >30% [2]. Recently, the World Health Organization has reclassified MDS, dividing RA into groups with one or several cytopenias, and changing the cut-off of transition to AML to 20%, thus eliminating the RAEBT category in the FAB scheme [3]. In addition, a separate diagnosis is made for patients with the syndrome of a deletion in the 5q chromosome (del(5q)) and <5% blasts, with normal or increased platelet counts. As with all classification (and reclassification) schemes, these are based on correlations with outcome, rather than the underlying biology of the disease.

**Linked Research Article**

This Research in Translation article discusses the following new study published in *PLoS Medicine*:

Ebert BL, Galili N, Tamayo P, Bosco J, Mak R, et al. (2008) An erythroid differentiation signature predicts response to lenalidomide in myelodysplastic syndrome. PLoS Med 5(2): e35. doi:10.1371/journal.pmed.0050035

Using gene expression profiling, Azra Raza and colleagues identified a molecular signature that predicts response to lenalidomide in patients without Chromosome 5q deletions, which suggests that these patients have a defect in erythroid differentiation.

The International Prognostic Scoring System uses a few simple variables to predict outcome (Tables 1 and 2) [4]. The percentage of marrow blasts, the presence of cytogenetic abnormalities (lumped into good, poor, and intermediate), and the number of cytopenias is used to derive a score that places patients into four risk categories. These outcomes are based on supportive care therapy. For low-risk disease, the median survival is 5.7 years; for intermediate risk–1, 3.5 years; for intermediate risk-2, 1.1 years; and for high risk, less than six months.

**Table 1:**
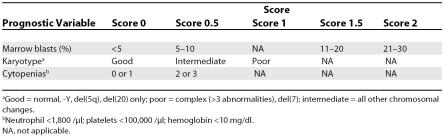
The International Prognostic Scoring System and Prognosis

**Table 2:**
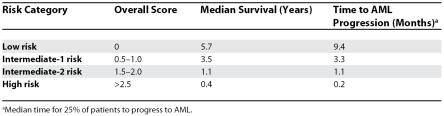
Risk Category and Prognosis

## The Biology of MDS

The biology driving MDS has been elusive, and has greatly undermined attempts to devise rational treatment options. Any biological model of MDS must explain several features of the disease: The paradox that early in disease patients appear to have increased proliferation *and* apoptosis; the variable natural history of disease, from progressive cytopenia to progression to AML; and the potential contribution of the stromal environment to disease maintenance and progression.

The genes and pathways involved in MDS are unclear [5]. Compared to AML, MDS tends to have more deletions of chromosomes (−5, −5q, −7, −7q, +8, 11q−, 20q−), implicating tumor suppressor genes, though the genes have not been identified [6]. Compared to AML, mutations or deletions involving tyrosine kinases, the ras pathway, and transcription factors are unusual (Figure 1). Recently, gene expression arrays have found that the progression of MDS from RA to advanced phases of MDS is associated with the aberrant expression of genes associated with proliferation (e.g., *c-myb*) and differentiation (e.g., *a, ß,* and *d-globin*) [7].

**Figure 1:**
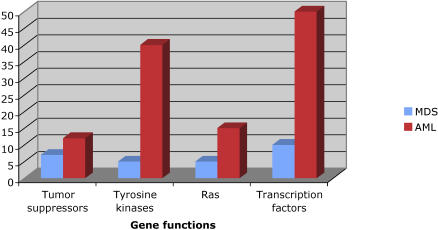
The Percentage of Cases of MDS and AML Harboring Specific Classes of Genetic Mutations

## Therapy for MDS

The quest for effective therapy for MDS has been disappointing. Secondary AML is especially problematic, since at this stage of MDS, the reserve of normal hematopoietic cells is marginal, and thus after chemotherapy, patients suffer from prolonged cytopenias since they lack the ability to muster normal hematopoiesis. Attempts to adjust and apply conventional AML therapy to MDS (e.g, low-dose Ara-C) have given uninspiring results.

There is a current wave of new approaches aimed at greater effectiveness that target other biological pathways. In general, all therapies work better with early disease (RA) compared with advanced stage disease. However, the only curative therapy is allogeneic transplantation. This procedure has the obvious limitation of needing an appropriately human leukocyte antigen-matched donor, and is fraught with complications from the high-dose conditioning regimen, infections, and graft-versus-host disease. Nonetheless, about 60% of patients transplanted with RA are disease-free at five years. If transplantation is deferred to advanced stage disease, survival falls to around 20% [8–10]. Recently, low-intensity transplants, which rely on the “graft-versus-leukemia” effect of the donor immune system against the malignancy, have been investigated. These transplants have quite similar results to the full myeloablative transplants, and can be given to an older population [11].

What about the vast majority of patients with MDS in whom transplantation is neither possible, nor preferred? A recent advance has been the introduction of the hypo-methylating agents 5-azacitidine and 5-aza-2′-deoxycytidine (decitidine) [12,13]. These agents cause a gradual change in the methylation status of the malignant clone, changing the expression of genes thought to be repressed from aberrant methylation-mediated silencing. Several large studies have shown that these agents are effective, yielding complete response rates from around 20% to 40%. Patients on these therapies appear to have an approximately 18-month prolongation of their survival compared to patients receiving the standard of care, with an approximately 2-fold decrease in the rate of progression to AML.

There are some data suggesting that MDS may be immunologically driven or modulated. Thus, attempts at immunoregulation using immunological agents such as cyclosporine and antithymocyte globulin are occasionally helpful. Likewise, antibody therapy directed at tumor necrosis factor receptors (Enbrel) shows some promise, causing correction in blood counts in about 25% of cases [14,15]. The thalidomide derivative lenalidomide (Revlimid) has shown considerable promise in low-risk MDS, especially in patients with del(5q). Lenalidomide appears to have immunomodulatory activity, as well as affecting cytokine and angiogenesis pathways. Lenalidomide appears remarkably selective for patients with del(5q), yielding complete cytogenetic responses in nearly 60% of patients. In addition, around 70% of patients had a decline in transfusion needs, while only about 25% of patients without the del(5q) enjoyed this benefit [16,17].

## In Search of Better Therapies for MDS

The recent excitement over modest response rates reflects long-standing frustration (if not desperation) in treating MDS. How do we find better therapies? A fundamental building block would be the understanding of *why* certain therapies work in a subset of patients. In this light, the work by Azra Raza and colleagues in this issue of *PLoS Medicine* is a strong step towards understanding the biology underlying treatment response in MDS [18]. The authors studied the gene expression signature associated with lenalidomide response in low-risk MDS. They first established the response signature in low-risk non-5q- MDS patients, and used a validation set of 5q- and non-5q-patients. They found that the gene expression response signature was enriched in genes involved in erythroid differentiation; as a biological confirmation, the authors demonstrate that the application of lenalidomide to primary non-MDS CD34^+^ cells promoted erythroid differentiation.

The study has a few limitations, but these do not retract from the findings or their significance. The study is fairly small, and thus may not be robust in regards to predicting response in a larger, more heterogeneous group. The arrays were performed on mononuclear samples, rather than purified CD34^+^ cells. While critics may cite this as a problem of deriving an expression signature from a heterogeneous sample, the potential advantage biologically is that the inclusion of the entire marrow sample studies the gene expression changes seen across the population, which may give a truer glimpse of the biology of the disease. From a practical side, the simpler sample is preferred, since if these studies are ever to be used for diagnostic purposes, an analysis of the restricted CD34+ population would be impractical.

The study is important on several fronts. First, the determination of a lenalidomide response signature may be important in selecting non-5q patients that will respond to that therapy. This will improve the MDS response rate to lenalidomide in the community by “enriching” for patients who should actually respond to the drug. It also points to an important pathway (erythroid differentiation) as a target for future drug development. Moreover, the approach outlined by the investigators should serve as a model for probing the genetics of response in other MDS therapy. One can imagine the day when small sets of genes can be used at diagnosis to guide the choice of therapeutic options.

**Six Key Papers in the Field**

**Kantarjian et al., 2007 [12] and Silverman et al., 2002 [13]** These two papers show the promise of the new demethylating agents, which are one of the few classes of drugs that appear to extend the natural history of MDS.

**List et al., 2006 [16]** This paper demonstrates the remarkable effect of lenalidomide on a specific subgroup of MDS.

**Vardiman et al., 2002 [3]** This paper updates the FAB classification system, and attempts to further define MDS risk groups based on clinical and pathological features.

**Greenberg et al., 1997 [4]** This paper provides a natural history prognosis based only on the number of cytopenias, chromosomal abnormalities, and blast count.

**Anderson et al., 1993 [8]** One of the first papers to give compelling evidence that allogeneic transplantation was potentially curative for MDS.

## Conclusion

The treatment of MDS is still distinctly suboptimal. New agents have brightened the picture, and studies such as that of Raza et al. promise to focus therapy on those patients who will most likely benefit. Nonetheless, substantial obstacles still confront us. We have little understanding of why MDS is age related; why patients progress to AML, and how to predict or prevent it; the relative roles of the MDS clonal cell and the stromal environment in determining response and progression; and how to add combinations of agents to better treat MDS. There is much to do, and the clock is running: we are all getting older, and MDS is waiting.

## Abbreviations

AML: acute myeloid leukemia
FAB: French, American, and British
MDS: myelodysplasia
RA: refractory anemia

## References

[pmed-0050040-b001] Heaney ML, Golde DW (1999). Myelodysplasia. New Engl J Med.

[pmed-0050040-b002] Bennett JM, Catovsky D, Daniel MT, Flandrin G, Galton DA (1982). Proposals for the classification of the myelodysplastic syndromes. Br J Haematol.

[pmed-0050040-b003] Vardiman JW, Harris NL, Brunning RD (2002). The World Health Organization (WHO) classification of the myeloid neoplasms. Blood.

[pmed-0050040-b004] Greenberg P, Cox C, LeBeau MM, Fenaux P, Morel P (1997). International scoring system for evaluating prognosis in myelodysplastic syndromes. Blood.

[pmed-0050040-b005] Pedersen-Bjergaard J, Andersen MT, Andersen MK (2007). Genetic pathways in the pathogenesis of therapy-related myelodysplasia and acute myeloid leukemia. Hematology Am Soc Hematol Educ Program.

[pmed-0050040-b006] Fenaux P, Morel P, Lai JL (1996). Cytogenetics of myelodysplastic syndromes. Semin Hematol.

[pmed-0050040-b007] Lee YT, Miller LD, Gubin AN, Makhlouf F, Wojda U (2001). Transcription patterning of uncoupled proliferation and differentiation in myelodysplastic bone marrow with erythroid-focused arrays. Blood.

[pmed-0050040-b008] Anderson JE, Appelbaum FR, Fisher LD, Schoch G, Shulman H (1993). Allogeneic bone marrow transplantation for 93 patients with myelodysplastic syndrome. Blood.

[pmed-0050040-b009] Scott B, Deeg HJ (2006). Hemopoietic cell transplantation as curative therapy of myelodysplastic syndromes and myeloproliferative disorders. Best Pract Res.

[pmed-0050040-b010] Deeg HJ, Storer B, Slattery JT, Anasetti C, Doney KC (2002). Conditioning with targeted busulfan and cyclophosphamide for hemopoietic stem cell transplantation from related and unrelated donors in patients with myelodysplastic syndrome. Blood.

[pmed-0050040-b011] Scott BL, Sandmaier BM, Storer B, Maris MB, Sorror ML (2006). Myeloablative vs nonmyeloablative allogeneic transplantation for patients with myelodysplastic syndrome or acute myelogenous leukemia with multilineage dysplasia: a retrospective analysis. Leukemia.

[pmed-0050040-b012] Kantarjian H, Oki Y, Garcia-Manero G, Huang X, O'Brien S (2007). Results of a randomized study of 3 schedules of low-dose decitabine in higher-risk myelodysplastic syndrome and chronic myelomonocytic leukemia. Blood.

[pmed-0050040-b013] Silverman LR, Demakos EP, Peterson BL, Kornblith AB, Holland JC (2002). Randomized controlled trial of azacitidine in patients with the myelodysplastic syndrome: a study of the cancer and leukemia group B. J Clin Oncol.

[pmed-0050040-b014] Deeg HJ, Gotlib J, Beckham C, Dugan K, Holmberg L (2002). Soluble TNF receptor fusion protein (etanercept) for the treatment of myelodysplastic syndrome: a pilot study. Leukemia.

[pmed-0050040-b015] Deeg HJ, Jiang PY, Holmberg LA, Scott B, Petersdorf EW (2004). Hematologic responses of patients with MDS to antithymocyte globulin plus etanercept correlate with improved flow scores of marrow cells. Leuk Res.

[pmed-0050040-b016] List A, Dewald G, Bennett J, Giagounidis A, Raza A (2006). Lenalidomide in the myelodysplastic syndrome with chromosome 5q deletion. New Engl J Med.

[pmed-0050040-b017] List A, Kurtin S, Roe DJ, Buresh A, Mahadevan D (2005). Efficacy of lenalidomide in myelodysplastic syndromes. New Engl J Med.

[pmed-0050040-b018] Ebert BL, Galili N, Tamayo P, Bosco J, Mak R (2008). An erythroid differentiation signature predicts response to lenalidomide in myelodysplastic syndrome. PLoS Med.

